# Hemorrhagic Fever with Renal Syndrome, Vietnam

**DOI:** 10.3201/eid1602.091204

**Published:** 2010-02

**Authors:** Vu Thi Que Huong, Kumiko Yoshimatsu, Vu Dinh Luan, Le Van Tuan, Le Nhi, Jiro Arikawa, Tran Minh Nhu Nguyen

**Affiliations:** Pasteur Institute, Ho Chi Minh City, Vietnam (V.T.Q. Huong, V.D. Luan, L. Nhi); Hokkaido University Graduate School of Medicine, Sapporo, Japan (K. Yoshimatsu, J. Arikawa); World Health Organization Technical Office, Ho Chi Minh City (L.V. Tuan); Vietnam Field Epidemiology Training Program Office, Hanoi, Vietnam (T.M.N. Nguyen)

**Keywords:** Hantavirus infection, Seoul virus, hemorrhagic fever with renal syndrome, virus, Vietnam, letter

**To the Editor**: Hantaviruses are primarily rodent borne and can cause hemorrhagic fever with renal syndrome (HFRS) in persons who inhale aerosolized excreta from infected rodents. The clinical characteristics of HFRS are fever, hemorrhage, and varying degrees of renal and hepatic dysfunction. Although HFRS is endemic primarily to Eurasian regions, there is serologic evidence of hantavirus infections in rodents and humans worldwide (*1*). Little is known about the occurrence of hantavirus infection in rodents or humans in Vietnam. One study found 5.4% prevalence of antibodies against Hantaan 76–118 and Puumala strains among residents of the Hanoi Metropolitan (*2*), whereas another study in southern Vietnam did not find evidence of hantavirus infection in humans (*3*). We describe autochthonous HFRS from Vietnam, possible reservoir hosts, and the follow-up investigation, which implies the presence of a strain of Seoul virus (SEOV).

The case-patient was a previously healthy 25-year-old nurse working in a referral hospital and residing in a semiurban district of Ho Chi Minh City. On September 23, 2008, she was admitted to the referral hospital with a history of high fever, chills, myalgia, nausea, vomiting, hematuria, and abdominal and lower back pain for 3 days. Physical examination showed a body temperature of >39°C, petechiae, mild dehydration and hypotension, with otherwise unremarkable vital signs. Hematologic tests showed 13,300 leukocytes/mm^3^, 167,000 thrombocytes/mm^3^, and hematocrit of 31%. Urinalysis showed grave hematuria (3+), proteinuria (2+), and leukouria (2+).

Three days after admission, acute renal failure with relative oliguria (0.85 L/24h) developed, as well as uremia (26.4 mg/dL), creatinemia (0.98 mg/dL), and abnormal liver function (aspartate aminotransferase 49 U/L and alanine transferase 60 U/L). The following day the patient had dyspnea and became agitated. Ultrasound examination showed pleural effusion, parietal pericardial effusion, peritoneal ascites, hepatomegaly, and renal thickness. Six days after admission, diuretic problems developed in the patient (3.7 L/24 h), her dyspnea resolved, and she became afebrile. Ten days after admission, the patient’s hematuria resolved, and renal and liver functions gradually recovered; she was discharged after 29 days of hospitalization.

Immunoglobulin (Ig) M and IgG against Hantaan recombinant nucleocapsid protein antigen were detected in the case-patient’s acute-phase and convalescent-phase serum samples, respectively, by ELISA (*4**,**5*). The presence of antihantavirus IgG was confirmed by immunofluorescent antibody (IFA) assay using whole hantavirus antigen and Western blot using hantavirus CL-1 strain (*6**,**7*). In further analysis, neutralization antibodies against SEOV strain SR-11 were detected by focus reduction neutralization test (*8*). The viral RNA, however, was not detectable in the acute-phase blood sample by reverse transcription–PCR (RT-PCR) (*9*). Other serologic tests were performed for dengue fever, typhoid fever, hepatitis B, and malaria; results of culture of blood and urine for bacteria were negative.

Following confirmation of the diagnosis, close contacts of the patient were investigated. Two family members of the patient did not have any symptoms compatible with HFRS; their serum samples were tested and found negative for antihantavirus IgG. Because the patient was a nurse, possible nosocomial transmission and sources were also investigated and excluded. On the basis of the patient’s strong history of exposure to rodents at home, further investigation focused on the domestic rodent population.

From October 14 through 16, 2008, 110 rodent traps were set within and surrounding the patient’s house. The total catch was 32 rodents, of which 16 were *Rattus norvegicus*, 7 *R. exulans*, 5 *R. argentiventer,* and 4 *Bandicota indica*. By using ELISA, IFA, and Western blot, antihantavirus IgG was detected in serum from 7 rats, of which 5 were *R. norvegicus*, 1 *R. argentiventer*, and 1 *B. indica*. Further analysis using RT-PCR identified 2 SEOV strains from *R. norvegicus and R. argentiventer* captured in the patient’s house. The M segment of 1 identified SEOV strain (24D1208) was sequenced and compared with 22 SEOV strains, 6 of which were from *R. norvegicus* rats captured in urban areas of North Vietnam. Phylogenetic analysis showed that this SEOV belonged to the Vietnamese SEOV genotype (Figure).

**Figure:**
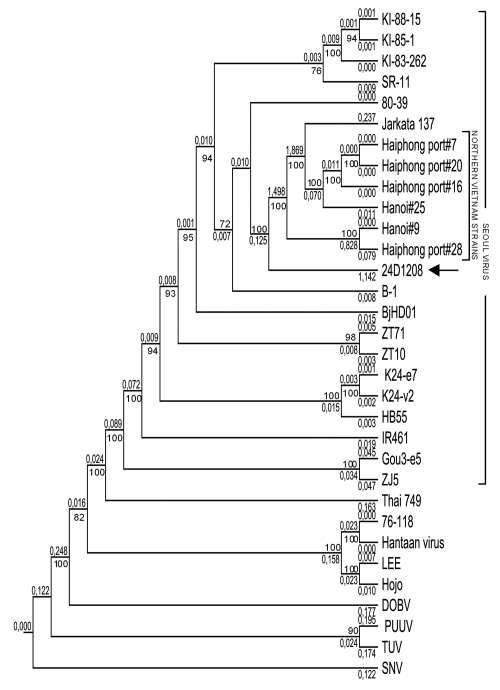
Phylogenetic tree (CLC-Combined Workbench 3) showing partial sequences of the medium segment (nt 810–2355). The newly identified Seoul virus (SEOV) was denoted as 24D1208 (arrow). The M segment sequences of the reference strains are: SEOV strains KI-88-15 (D17594), KI-85-1 (D17593), KI-83-262 (D17592), SR11 (M34882), 80–39 (S47716), Jakarta137 (AJ620583), Haiphong port #7 (AB355728), Haiphong port #20 (AB355730), Haiphong port #16 (AB355729), Hanoi #25 (AB355733), Hanoi #9 (AB355732), Haiphong port #28 (AB355731), B-1 (X53861), BjHD01 (DQ133505), ZT71 (EF117248), ZT10 (DQ159911), K24-e7 (AF288652), K24-v2 (AF288654), HB55 (AF035832), IR461 (AF458104), Gou3-e5 (AF288650), and ZJ5 (FJ811839); Thailand virus strain 749 (L08756); Hantaan virus strains 76–118 (M14627), Hantaan (NC005219), LEE (D00377) and Hojo (D00376); Dobrava virus (DOBV) strain Dobrava (L33685); Puumala virus strain Sotkamo (X61034); Tula virus (TUV) strain Tula/Moravia/5302v/95 (Z69993); and Sin Nombre virus (SNV) strain NMH10. The numbers at the nodes are bootstrap confidence levels for 1,000 replications. Only bootstrap support values >70% are shown.

We describe a clinical case of hantavirus infection and its potential rodent reservoir occurring in Vietnam. The clinical manifestations of the case-patient were compatible with SEOV infection, which is responsible for a moderate form of HFRS (*10*). Also, HFRS caused by SEOV occurs in urban rather than rural areas, unlike other hantavirus infections. Our epidemiologic findings were compatible with other studies indicating the source of infection was the case-patient’s home, the only place where she had a history of exposure to rodents. Although viral RNA could not be obtained from the case-patient for genotyping, the genomic comparison of the viral strains from rodents captured in the case-patient’s home and elsewhere in Vietnam suggested that the source of infection was local rodents. This report provides additional evidence that hantavirus infection is a worldwide problem and is likely underdiagnosed in Vietnam and other countries where simple standardized laboratory diagnostics are not widely available.
